# A Symptom of Arsenicum

**Published:** 1892-01

**Authors:** Ella M. Tuttle

**Affiliations:** New Berlin, N. Y.


### A SYMPTOM OF ARSENICUM.

ELLA M. TUTTLE, M. D., NEW BERLIN, N. Y.

I wish to say a few words in regard to a symptom of Ars., upon which it seems to me sufficient stress is not laid. I have found it as much of a keynote for that remedy as "frequent thirst for small quantities of water," or "burning relieved by heat." I refer to the symptom, "pains like hot needles piercing." It has often led me to Ars. as a remedy, when otherwise I would have been very much in doubt. Let me give one case in illustration:

Mrs. ——, aged seventy-six, naturally very bright and active. For two years had been afflicted with an ulcer over the outer malleolus of each ankle. These had been healed several times by her allopathic physician, but each time would soon open again. When I first saw her the ulcers were each about the size of a silver dollar, and the entire ankle on the left foot was red and angry looking. The chief thing that she complained of during the day was the stiffness of the ankle joints, but at night, when she became warm in bed, there was almost intolerable itching of the skin around the ulcers, momentarily relieved by scratching, sometimes relieved by hot applications, sometimes by cold. The base of the ulcer was red and raw-looking, and there was a slight watery discharge. Her general health was good. I gave her Merc.$^{30}$, but with no relief. At my next call I obtained no further light on the case till, just as I was leaving, she mentioned that there was occasionally a little pain in the ankles "like hot needles sticking through them." How many times we get our most valuable symptom after we have started to go. Well, I had seen that symptom mentioned in THE HOMŒOPATHIC PHYSICIAN as an indication for Arsenicum, so I gave her that remedy. To my delight she began to improve from that time, the pains disappeared, the inflammation subsided, and the ulcers slowly healed. This was over a year ago, and as there has been no return of the ulcers, I think that I am safe in pronouncing it a cure. I gave one dose of Arsenicum a week using the 30th dil. at first, and afterward the 200th.

